# Supplementary material for: Surface Microbiome Profiling of Dental Elevators Using Third‐Generation Sequencing: Implications for Infection Control in Dental Practice
Source: Microbiologyopen. 2025 Dec 8;14(6):e70178. doi: 10.1002/mbo3.70178 (PMC12685768; doi:10.1002/mbo3.70178)
Supplement: Supplementary file 1 — customer_backup. [file MBO3-14-e70178-s001.zip › customer_backup/customer_backup/taxa_summary/krona/samples/M12.Krona.html]

Javascript must be enabled to view this page.

members
magnitude
magnitudeUnassigned

M12.krona

57524

57516

63

63

63

63

63

10

7

7

7

11

13

8

7861

106

106

1

1

1

102

7

4

2

1

6

5

1

19

19

44

42

2

1

1

23

1

20

2

2

2

3

3

3

76

76

76

76

76

1148

69

69

48

48

5

5

12

12

4

4

168

25

25

25

21

21

21

5

5

5

7

7

7

7

7

7

103

103

103

251

7

7

7

3

3

3

40

40

40

2

2

2

173

173

173

26

26

26

2

2

2

2

658

46

43

43

3

3

146

146

1

7

138

246

5

5

241

241

220

15

1

14

197

2

195

8

5

3

2413

1465

3

3

3

661

480

1

169

5

47

85

131

42

1

1

16

11

5

82

4

2

76

50

21

17

12

32

32

801

55

55

3

3

739

739

4

4

948

453

453

50

396

7

495

4

4

491

491

4052

1

1

1

1

1

1

1

1

4

4

4

4

9

9

9

9

3

3

3

3

43

43

43

15

5

9

13

1

43

43

3

3

1

1

4

2

2

1

1

3

3

2

2

5

2

1

2

10

8

2

3

3

9

9

2

2

484

81

19

19

2

1

1

8

8

52

52

403

28

7

8

13

4

4

329

1

10

1

1

107

11

10

3

23

20

9

1

59

1

1

6

49

2

7

7

16

16

24

24

2

2

15

15

13

1

12

2

2

39

39

39

2

18

11

8

150

1

1

1

61

61

6

29

19

7

88

78

2

76

7

7

3

3

223

97

19

19

77

77

1

1

20

20

20

2

2

2

3

3

3

4

4

4

97

3

3

17

17

77

77

78

78

4

4

12

2

1

9

49

8

6

6

19

10

1

1

6

6

5

5

1

1

16

1

1

1

15

15

13

2

2943

42

4

4

1

1

3

3

5

5

25

3

22

4

4

2145

4

4

1

1

1

1

270

270

2

2

25

25

326

326

1516

4

1025

19

366

102

172

155

152

1

2

17

17

548

1

1

10

10

12

2

10

1

1

13

13

3

3

3

3

481

50

431

11

11

3

3

9

9

1

1

4

4

4

32

23

23

9

9

66

66

66

66

4

30

26

6

5

4

4

4

4

4

1

1

1

1

1

413

413

413

413

413

413

221

221

221

221

220

220

1

1

71

71

71

70

70

15

55

1

1

1

34

6

6

4

4

4

2

2

2

28

28

5

5

5

23

23

23

356

304

1

1

1

1

3

3

2

2

1

1

300

4

4

4

1

1

1

1

1

1

2

2

2

292

292

292

52

17

17

17

17

35

10

10

10

4

4

4

2

2

2

5

5

5

14

14

14

72

65

65

65

40

40

18

4

14

7

7

3

3

1

1

1

2

2

2

4

1

1

1

1

3

3

3

3

9

9

9

9

8

5

3

1

1

18

18

18

18

18

18

615

310

21

21

21

21

289

92

81

74

7

2

2

1

1

8

8

197

32

32

20

20

7

7

138

138

145

55

55

55

10

38

6

1

4

3

3

3

1

1

1

86

86

1

1

31

12

19

27

4

23

24

8

1

15

3

3

11

11

11

11

11

76

76

76

76

7

26

3

1

19

20

73

73

6

6

6

55

55

55

12

12

12

755

2

2

2

2

2

16

2

2

2

2

6

1

1

1

5

5

5

1

1

1

1

5

2

2

2

3

3

3

2

2

2

2

8

8

1

1

1

4

4

4

3

3

3

729

729

502

502

502

12

12

12

26

26

26

50

50

50

35

35

35

18

18

18

86

86

86

2

2

1

1

1

1

1

1

1

1

8

8

8

4

4

4

1

1

1

3

3

3

551

14

14

14

14

14

121

121

121

7

7

72

72

37

1

36

5

4

1

416

416

416

89

87

2

316

8

166

8

134

7

7

4

3

1

389

120

120

120

120

120

1

1

1

1

1

268

143

129

80

26

46

8

49

3

25

21

14

14

14

5

5

2

2

3

3

112

26

5

5

1

1

20

4

16

30

30

5

25

56

56

13

43

8

8

3

3

1

1

4

1

3

38

38

38

15

15

15

23

11

4

7

12

1

2

9

101

62

12

12

4

4

8

6

2

50

21

21

21

18

18

18

1

1

1

7

7

7

3

3

3

18

18

18

18

18

21

13

13

7

7

6

6

8

8

8

1

7

33046

14683

475

36

36

36

348

3

3

27

26

1

10

10

308

1

306

1

91

91

91

232

232

154

1

153

74

74

4

4

23

23

4

4

19

19

55

55

24

2

1

21

31

3

4

24

2

1

1

1

1

1

1

4439

4439

2

2

232

24

9

89

8

102

88

88

2

2

119

119

6

6

19

5

5

4

1

4

586

5

372

65

144

3290

67

3

9

1

1

14

1

7

298

62

6

1

927

685

1018

1

2

27

151

4

1

2

2

95

11

2

82

22

1

1

1

21

21

21

7746

212

16

16

44

36

8

8

8

17

13

4

127

42

9

76

14

14

14

335

21

21

211

82

7

3

62

5

47

5

103

103

1

1

1

4

4

4

4

4

4

5

5

5

6

6

6

4676

5

5

283

276

7

185

179

6

2693

2693

1071

20

141

822

29

8

1

50

37

37

13

13

367

3

39

6

27

4

5

7

4

1

32

15

224

22

22

135

3

3

4

4

102

102

12

12

14

14

11

11

11

2

2

2

9

9

9

121

9

1

2

6

33

33

79

5

37

13

24

67

55

54

1

12

12

535

18

5

13

36

36

114

114

39

34

5

2

2

34

34

18

18

5

5

269

54

215

32

32

32

1577

377

377

15

15

5

5

33

27

1

5

570

24

46

7

178

37

103

62

9

83

10

11

21

21

21

8

2

9

2

2

2

3

3

530

530

242

7

3

3

4

4

235

120

8

2

61

49

31

4

27

20

5

2

13

14

14

50

1

49

13

13

13

13

26

26

20

20

6

6

3

3

3

3

3

3

3

3

74

74

74

71

3

3

3

3

3

154

154

1

1

39

4

35

58

17

11

30

26

5

11

1

4

2

3

2

2

27

10

17

1

1

3

3

3

3

297

14

14

14

168

168

168

2

2

2

12

12

12

1

1

1

8

8

8

5

5

5

87

87

87

871

871

5

5

44

44

1

1

22

8

3

7

3

1

16

4

12

12

12

727

18

278

431

44

44

18363

12

12

12

4

8

15

14

14

14

1

1

1

1630

1

1

1

1

1

1

1125

373

373

8

8

131

131

613

613

3

3

1

2

9

9

9

377

377

377

114

114

114

11204

104

104

104

34

7

7

27

27

5

1

1

4

4

2

2

2

9

9

9

64

64

64

6656

1

1

1

1

5224

5224

535

13

4

60

458

895

2

64

8

5

29

430

34

48

11

112

1

10

41

47

2

51

126

17

17

1

1

75

69

6

17

17

15

15

1

1

7

7

7

70

1

1

2

2

1

1

53

1

2

50

13

10

3

117

117

117

3955

24

1

4

19

11

11

523

523

84

1

83

44

44

161

41

120

151

114

10

27

604

122

482

4

4

5

5

6

6

2121

2121

216

216

1

1

55

24

24

31

1

30

61

58

34

24

10

20

20

4

4

3

3

3

9

9

9

9

1

1

1

1

3

3

3

3

109

109

109

8

6

3

2

6

9

75

3070

544

193

193

51

1

50

294

294

2

2

4

1

3

2526

855

855

6

2

1

3

19

14

5

30

13

13

4

1616

477

1

6

474

131

33

8

29

42

415

3

3

3

3

2

2

2

2

2109

2

2

2

2051

2051

20

1948

83

1

1

1

55

7

6

1

1

1

5

5

42

2

1

39

5

5

5

5

47

47

40

7

14

19

4

4

3

3

13

13

13

13

4

4

4

4

59

59

59

59

1

1

1

1

6

6

6

6

4

4

4

4

4

4

430

295

295

1

1

1

294

13

13

12

12

21

21

34

34

41

41

29

10

19

144

101

33

10

135

3

2

2

2

1

1

1

78

27

1

1

3

1

2

23

7

3

13

2

2

2

35

2

1

1

33

14

11

8

14

2

2

12

12

9

9

9

9

45

45

45

3

19

23

26

26

26

26

26

3

4

19

122

14

1

1

1

1

7

5

5

5

2

2

2

6

6

6

6

3

2

2

2

2

1

1

1

1

36

2

2

2

2

19

19

19

19

6

6

6

6

5

5

1

1

4

4

4

3

3

3

1

1

1

69

69

69

69

69

8598

4209

7

7

3

2

1

1

1

3

1

1

1

1

1

1

1

32

2

2

2

1

1

1

29

29

29

578

30

10

5

5

16

2

14

4

4

2

2

2

534

4

4

375

375

155

33

122

12

3

3

4

3

1

4

4

1

1

13

13

13

3

10

2453

2453

31

30

1

2

2

86

14

41

2

2

1

5

2

1

3

4

2

1

8

107

37

70

27

27

51

46

5

4

4

353

131

9

212

1

58

50

8

20

1

5

13

1

13

13

241

19

214

8

85

85

23

23

3

3

8

2

6

50

50

5

5

125

8

82

35

4

4

3

3

40

40

4

4

5

5

3

3

10

6

4

45

16

29

4

4

1043

5

463

1

14

1

196

321

21

19

2

647

647

647

3

3

5

4

628

4

17

6

6

6

3

3

3

8

8

8

1

1

1

1

14

7

7

7

7

7

7

446

255

3

3

9

2

7

15

10

2

2

1

1

1

8

1

1

6

72

72

2

2

12

1

4

2

5

49

49

27

4

23

25

25

32

32

51

51

51

5

2

2

3

3

115

5

3

2

29

13

8

4

4

42

42

28

3

3

21

1

8

8

3

2

1

18

1

1

13

10

3

4

4

2

2

2

4332

1160

314

161

161

58

2

56

37

37

21

21

2

2

5

5

30

27

1

1

1

846

846

5

54

10

6

1

370

278

19

56

7

5

17

18

483

155

22

22

96

30

57

9

1

1

26

16

10

10

10

328

78

53

25

13

13

4

4

7

7

1

1

84

15

69

61

61

8

8

53

27

26

13

3

10

4

4

2

2

1

1

1

1

13

12

3

3

6

6

3

3

1

1

1

5

5

5

5

332

332

1

1

279

49

99

2

3

1

7

21

10

3

8

41

3

2

2

15

5

1

2

3

2

11

11

41

5

21

15

9

1

1

1

2

2

2

6

6

6

2110

2110

2110

2091

19

215

7

7

6

1

2

1

1

1

1

40

36

2

18

2

2

5

7

4

4

15

15

15

151

14

12

1

1

3

3

43

22

3

11

7

20

19

1

11

11

39

39

1

1

10

1

9

1

1

9

9

4

4

4

2

2

32

32

4

3

3

1

1

20

6

6

14

14

8

8

8

1

1

1

1

1

24

24

24

24

24

7

7

7

7

7

3

4

161

161

161

161

161

74

87

187

142

2

2

2

2

140

139

139

139

1

1

1

45

38

38

19

17

2

4

4

15

5

10

7

7

7

7

3298

3286

966

4

4

4

936

11

10

1

12

3

5

4

25

6

11

6

2

27

4

21

2

5

5

2

2

417

4

25

13

10

16

328

5

5

11

149

1

79

60

5

4

3

3

26

12

3

11

90

90

102

35

67

67

67

26

26

1

14

11

688

2

2

2

686

686

686

1389

41

41

2

11

14

1

2

3

4

4

40

1

1

39

3

2

34

381

381

1

75

169

54

4

5

48

2

1

1

20

1

180

28

28

152

50

31

22

24

25

307

241

241

16

16

50

50

440

12

12

30

30

12

12

386

386

11

11

7

1

6

4

4

232

9

2

2

5

3

2

2

2

6

3

3

2

2

1

1

16

2

2

14

1

9

4

3

3

3

55

41

41

8

8

6

2

3

1

143

7

7

60

7

2

37

4

10

4

4

11

6

5

61

1

12

1

3

32

2

2

1

2

5

4

4

4

4

4

8

8

8

8

8

55

55

55

6

6

6

10

10

10

31

31

31

8

8

8

8

1

1

1

1

1

1

5

5

5

5

5

5

2

2

2

2

2

2
